# Learning to Learn: Theta Oscillations Predict New Learning, which Enhances Related Learning and Neurogenesis

**DOI:** 10.1371/journal.pone.0031375

**Published:** 2012-02-10

**Authors:** Miriam S. Nokia, Helene M. Sisti, Monica R. Choksi, Tracey J. Shors

**Affiliations:** Department of Psychology, Center for Collaborative Neuroscience, Rutgers University, Piscataway, New Jersey, United States of America; Louisiana State University Health Sciences Center, United States of America

## Abstract

Animals in the natural world continuously encounter learning experiences of varying degrees of novelty. New neurons in the hippocampus are especially responsive to learning associations between novel events and more cells survive if a novel and challenging task is learned. One might wonder whether new neurons would be rescued from death upon each new learning experience or whether there is an internal control system that limits the number of cells that are retained as a function of learning. In this experiment, it was hypothesized that learning a task that was similar in content to one already learned previously would *not* increase cell survival. We further hypothesized that in situations in which the cells are rescued hippocampal theta oscillations (3–12 Hz) would be involved and perhaps necessary for increasing cell survival. Both hypotheses were disproved. Adult male Sprague-Dawley rats were trained on two similar hippocampus-dependent tasks, trace and very-long delay eyeblink conditioning, while recording hippocampal local-field potentials. Cells that were generated after training on the first task were labeled with bromodeoxyuridine and quantified after training on both tasks had ceased. Spontaneous theta activity predicted performance on the first task and the conditioned stimulus induced a theta-band response early in learning the first task. As expected, performance on the first task correlated with performance on the second task. However, theta activity did not increase during training on the second task, even though more cells were present in animals that had learned. Therefore, as long as learning occurs, relatively small changes in the environment are sufficient to increase the number of surviving neurons in the adult hippocampus and they can do so in the absence of an increase in theta activity. In conclusion, these data argue against an upper limit on the number of neurons that can be rescued from death by learning.

## Introduction

Since the discovery of adult neurogenesis in the hippocampus, there have been many theories about its role in learning [1]–[4]. We have previously reported that the new neurons are involved in associating events across time [5]. We have also shown that this type of learning keeps 1–2 weeks old new neurons from dying prematurely [6], [7]. However, only tasks that are relatively complex and/or difficult to learn will rescue the new neurons from death [8]. Mere exposure to training or rapid learning does not have the same beneficial effects. Moreover, retraining or relearning the same task after extinction does not rescue newly-generated hippocampal cells [7]. In addition, training and/or learning does not change the number of cells produced during or after the training experience [7], and the number of new cells present at the time of training does not predict how well an animal will learn [7], [9]. To summarize, new neurons in the hippocampus are especially responsive to new learning experiences and not necessarily to re-learning or rehearsal of already-acquired information. In addition, the effects are evident in terms of survival and not proliferation.

Animals in the natural world encounter new learning experiences all the time. Many of these situations can be rapidly assessed because of associations and/or skills that have been previously acquired. The first goal of this experiment was to determine whether we could detect an upper limit on the number of cells that can be rescued from death by successive learning opportunities. If new cells only survive in response to an entirely new learning opportunity, this would indicate the presence of a mechanism for incorporating and maintaining an optimal (and perhaps minimal) number of new neurons into the existing circuitry of the dentate gyrus. It is not known what the new cells might do once they are rescued and how their rescue influences future cohorts of new neurons as animals continue to respond to and learn from their environment. Here we hypothesized that learning one associative learning task would enhance the survival of one cohort of new neurons and those neurons would then mature and be used to learn a second, similar task. As a consequence, we expected that learning a second, related task would be easier and would *not* rescue a new cohort of neurons generated between the tasks, at least not in animals that learned the first task well.

To survive, new adult-born cells in the hippocampus must be integrated into functional neural networks. The mechanism by which this occurs is not known. One possibility is that the new cells are recruited as thousands of cells fire in synchrony. The most prominent and well-characterized electrophysiological rhythm in the hippocampus is theta, which oscillates between 3–12 Hz (for review see [10]). Spontaneous hippocampal theta activity can be divided into two types [11]. While awake, Type 1 theta is associated with voluntary locomotor activity such as running and its generation is resistant to systemically administered atropine (non-cholinergic). Type 2 theta is related to a state of alert immobility and is abolished by systemically administered atropine (cholinergic). Like neurogenesis, theta activity is often considered part of the neuronal mechanism(s) used to associate events across temporal intervals [12], [13], such as those that occur during classical conditioning procedures. In fact, theta activity in the hippocampus increases during eyeblink conditioning [14], [15] and synchronizes with theta activity in the cerebellum, the structure essential for learning to occur [16], [17], possibly indicating enhanced information flow between the two structures. Moreover, if training is targeted to occur when theta (specifically Type 2) is prominent, the animals learn faster [18], [19]. The predictive relationship between theta recorded before training and subsequent learning performance is strongest early in training, when the animal is acquiring the basic association between temporally related events [14], [20].

The second goal of this experiment was to determine whether theta activity induced during the learning process would correlate with the number of cells that later survived in the dentate gyrus. Based on previous studies, we hypothesized that spontaneous hippocampal Type 2 theta activity would predict the rate and/or degree of learning and that the conditioning stimuli would induce a theta-band response that would change in response to training, especially early in the learning process [14], [20]. We further speculated that the anticipated increase in induced theta activity would recruit new hippocampal cells into functional neural assemblies and thus rescue them from premature death. If this hypothesis proved to be correct, an increase in theta should accompany all training conditions that rescue new neurons from death and should not occur during those that do not.

**Figure 1:**
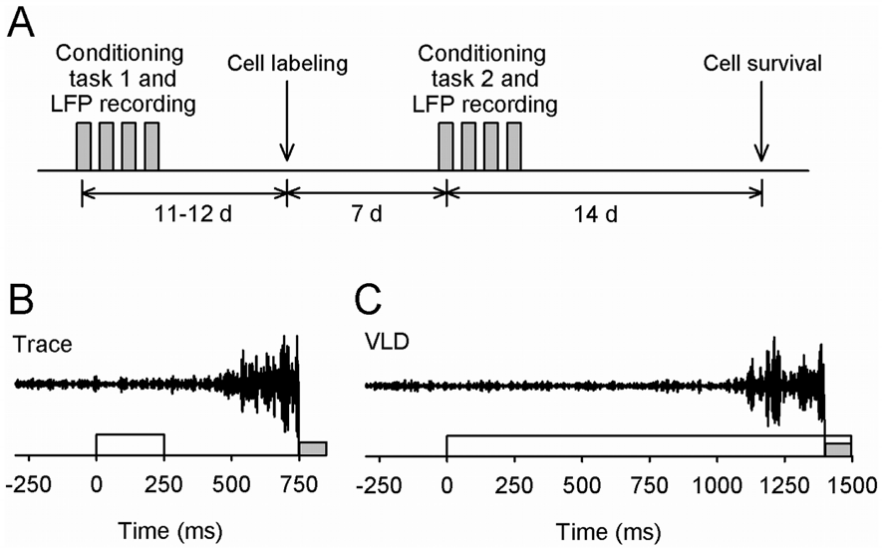
Schematic depicting the timeline of the experiment (A) and the two training protocols used (B and C). Rats were trained for 4 days with either trace (B) or very-long delay eyeblink conditioning (C). Local-field potentials (LFPs) from the dentate gyrus were also recorded during training. Several days after training on the first task had ceased BrdU was injected i.p. to label dividing cells. A week after the BrdU injection, rats were trained again, but now with the other task, while recording LFPs. The order of the tasks was counterbalanced. All rats were sacrificed 21 days after the BrdU injection to examine the number of surviving immature cells in the dentate gyrus. In B and C, the white bar indicates the white-noise conditioned stimulus and the grey bar indicates the stimulation to the eyelid used as an unconditioned stimulus. Representative learned responses obtained from single-trial electromyogram recordings are also presented.

To test these hypotheses, adult male Sprague-Dawley rats were trained on two associative learning tasks that differed in their temporal organization: trace and very-long delay eyeblink conditioning (see Fig. 1). Both tasks depend on the hippocampus for learning [21] and learning either task rescues new neurons from death [22]. Hippocampal local-field potentials were recorded before, during and after training. To assess cell survival in response to training on the second task, we labeled cells that were generated after training on the first task. One week later, when most of the new cells begin to die in the absence of learning, the animals were trained on the second task.

## Materials and Methods

### Ethics Statement

The experiments were designed to fully comply with the rules and regulations set forth by the PHS Policy on Humane Care and Use of Laboratory Animals and the Guide for the Care and Use of Laboratory Animals and were approved by the Rutgers University Animal Care and Facilities Committee (P.I. Tracey J. Shors, protocol nr. 98-018).

### Subjects

Twenty-eight Sprague-Dawley male rats bred in the Department of Psychology at Rutgers University were used. They were 60–75 days old and weighed 300–400 g at the beginning of the experiment. Rats were single housed during the experiment and food and water was available ad libitum. Lights were on for 12 h a day starting at 7.00 am. All experimental procedures were carried out during the light portion of the day.

### Surgery

Surgery was conducted in order to implant electrodes for assessing electrophysiological oscillatory activity (local-field potentials, LFPs) in the hippocampus as well as electrodes for measuring electromyographic (EMG) activity of the eyelid during eyeblink conditioning. Rats were anesthetized using an intraperitoneal (i.p.) injection of sodium pentobarbital (60 mg/kg; Nembutal, 50 mg/ml, Lundbeck Inc., Deerfield, IL, USA). Atropine (0.54 mg/ml, Vedco Inc. St. Joseph, MO, USA) was also injected i.p. to keep the rat's airways clear during surgery. The head was shaved and the rat was secured to a stereotaxic device (David Kopf Instruments, Tujunga, CA, USA) using blunt ear bars. A local analgesic (Marcaine, bupivicaine, 2.5 mg/ml, Hospira Inc., Lake Forest, IL, USA) diluted in saline (1.25 mg/ml) was injected (4×0.03 ml) subcutaneously to the site of the incision. An incision was made to reveal both the bregma and the lambda. Four skull screws were implanted and connected in pairs to serve as reference and ground for LFP recordings. Two electrodes were lowered into the right hippocampus aiming at the dentate gyrus (DG). The coordinates used were 3.5–4.2 mm posterior to bregma, 1.5–2.0 mm lateral to bregma and 3.4–3.8 mm below bregma. Electrodes were made of Formvar insulated nichrome wire (bare diameter 50 microns, A-M Systems Inc., Carlsboro, WA, USA). Next, two bipolar electrodes were implanted through the upper right eyelid. These electrodes were made of stainless steel wire insulated with Teflon (bare diameter 127 microns, A-M Systems Inc.). Finally, the whole construction was cemented in place using dental acrylic mass anchored to the skull via the skull screws. As soon as awake, the rats were given a 1-ml oral dose of acetaminophen (Children's Acetaminophen, 32 mg/ml, Rite Aid Corp., Camp Hill, PA, USA) and returned to their home cages and monitored daily for 5 days or until they had fully recovered.

### Conditioning procedure

The experimental set-up is depicted in Figure 1. Rats were acclimated to the conditioning chamber by placing them into the chamber for 1 h with the headstage secured but without any stimulation. On the next day, animals were presented with 10 pulses of white noise (83 dB, 250 ms). Responses to these presentations were counted and used to determine whether any of the animals were emitting eyeblink responses to the noise stimulus before any conditioning had occurred. Then animals began training on one of two associative learning tasks; either trace (Fig. 1B) or very-long delay (VLD, Fig. 1C) eyeblink conditioning. Both tasks included a white noise as a conditioned stimulus (CS) and a 100-ms periorbital shock (0.65 mA) as an unconditioned stimulus (US). During trace conditioning, the CS duration was 250 ms and a temporal gap of 500 ms was left between the CS offset and the US onset. During VLD conditioning, there was no gap. Instead, a CS of 1500 ms overlapped and co-terminated with a US of 100-ms. Rats were presented with 200 trials a day with an inter-trial interval of 25±5 s. Rats were trained for a total of 4 sessions on consecutive days (total of 800 trials per task). During training, both EMG from the upper eyelid and LFPs from the hippocampus (dentate gyrus) were recorded. At the beginning and end of each session, a 5-min stimulus-free period of spontaneous activity was also recorded.

### Cell labeling

All animals in the current experiment were injected i.p. once with bromodeoxyuridine (BrdU, 200 mg/kg; concentration: 15 mg/ml in 0.9% physiological saline). The injection was given 4–5 days after training on the first task was completed, that is, 7 days before training on the second task was started (see Fig. 1A). In the ideal case, the size of a selected cohort of new cells in each animal's hippocampus could be examined at multiple time points over the course of an experiment. In reality, because cell counts can only be retrieved post-mortem, this is not possible. However, to better interpret results from our current study, we can utilize the results of several previous studies suggesting that eyeblink classical conditioning does not affect the number of cells generated in the hippocampus during training but rather enhances the survival of cells present at the time of training. For example, there is no learning-induced increase in cell number if BrdU is injected and animals are trained 30 minutes later [7], nor if they are injected in the middle of the training procedure [6]. Note that the timing of the BrdU injection in the current experiment is identical to that used in our recent study [7] reporting no persistent increase in cell production in response to training and, moreover, reporting that retraining on or relearning the same task does not increase the survival of an entirely new cohort of BrdU-labeled cells. In addition, there is no observable increase in the number of new cells made after training. For example, no increase in cell number occurred if BrdU was injected 7 days after training and survival assessed 7 days thereafter [7]. However, as noted, the number of surviving cells does increase if the learning experience occurs when the cells are about 1–2 weeks of age [7]. To conclude, in the current experiment, we did not replicate all of the findings reviewed above but rather focused on the effects of learning on a cell cohort that has not yet been studied.

### Recording and data analysis

The EMG signal was band-pass filtered between 300 and 500 Hz (1700 Differential AC amplifier, A-M Systems Inc.). The LFPs were filtered between 1 and 500 Hz (PGA16, MultiChannel Systems, Reutlingen, Germany) and pre-amplified 10×. All signals were sampled at a rate of 2000 Hz and recorded continuously (Digidata1440 and AxoScope, Molecular Devices Corp., Sunnyvale, CA, USA). Matlab (The MathWorks Inc., Natick, MA, USA), PASW and SigmaPlot (SPSS Inc., Chicago, IL, USA) were used for data analyses.

To assess the percentage of eyeblinks in response to the CS, the amplitude envelope of the EMG signal was first derived using the Hilbert transformation. The mean (M) and the standard deviation (SD) of the signal during a 250-ms pre-CS period was then obtained. For each trial, the threshold for a conditioned response (CR) was set at M+4*SD. If this threshold was smaller than 0.5 V, 0.5 V was used as a threshold value instead. To be classified as a CR, the EMG signal had to exceed the threshold during a 250-ms period immediately preceding the US onset for at least 10 milliseconds. We chose to evaluate the 250-ms period before the US because it is a conservative estimate of conditioning. By using this more conservative threshold, we attempted to detect the most accurate performance in individual rats. It is not cost-efficient to close the eyelid earlier than a couple hundred milliseconds before impact, because other (visual) information potentially relevant for survival might be missed. Also, in our experience rats have a tendency to blink in response to the onset of the CS, especially early in training. By using the time window specified above we eliminated the possibility that these nonspecific blinks would erroneously be classified as conditioned responses. However, it is noted that the entire 500 ms range was also analyzed and correlated with the 250 ms range (r = 0.9). Animals were considered to have learned if they emitted at least 60% CRs during at least one 100-trial block. Rate of acquisition was quantified as the number of trials to reach the 10th CR and as the number of trials to reach the 60% learning criterion.

As mentioned, in addition to recordings made during the conditioning session, a 5-min stimulus-free period of spontaneous activity was also recorded at the beginning and end of each conditioning session. To assess the relationship between spontaneous levels of hippocampal theta and subsequent learning, at least 25 artifact-free 3-s epochs per animal were extracted from the LFPs recorded immediately before training on the first and the second task was started (1^st^ session of each task, 5-min recordings, no stimuli). Sweeps with artifacts most commonly caused by rapid large scale movements were automatically rejected from the analysis by simple amplitude thresholding. To determine the relative power of hippocampal theta activity [theta/(delta+theta)], fast Fourier transform (FFT) was calculated on the LFPs. From the FFT results, the relative power of Type 2 theta was calculated as the ratio between 3.5–5.5 Hz and 1.5–12.5 Hz activity and the relative power of Type 1 theta as the ratio between 5.5–12.5 Hz and 1.5–12.5 Hz activity.

To examine theta activity in response to the CS across training, theta ratios immediately prior to the CS and after the CS were derived. Data from all animals, regardless of the order of the conditioning tasks, were combined because previous studies indicate an increase in theta activity both during delay as well as during trace conditioning [14], [15]. Pre-trial spontaneous theta ratio was determined from a 500-ms time period immediately preceding the CS onset. CS-induced theta ratio was determined from a 500-ms time period starting 250 ms after the CS onset, thus avoiding the effect of immediate event-related potentials. Trials with artifacts most commonly caused by rapid large scale movements were automatically rejected from the analysis by simple amplitude thresholding. Initially, we analyzed Type 1 and Type 2 theta bands separately as described for spontaneous recordings above. However, the final analyses were based on a theta ratio determined from a single, wide band including both Type 1 and Type 2 theta (3.5–12.5 Hz/1.5–12.5 Hz). The comparison of the results using separate vs. combined theta bands showed that the hippocampal oscillatory response induced by the CS had a frequency of around 6–10 Hz which would be categorized as Type 1 theta. As noted, spontaneous Type 1 theta has been associated with voluntary locomotor activity in rats [11]. In the present experiment, responses to the CS were within the Type 1 theta frequency band and yet no gross motor movements were detected by the experimenter. Indeed, any major motor activities were reflected in the LFP recording as large-amplitude artifacts and would have been automatically excluded. If something, the rats expressed a decrease in movement along with anticipatory freezing behavior in response to training. As such, they remained immobile for the better part of all recordings. Therefore, it would be misleading to characterize the CS-induced theta-band response as a Type 1 theta response. Hence, results based on the wide-band theta ratio are reported and the response is simply referred to here as theta-band response.

### Statistical analyses

Repeated measures analysis of variance (ANOVA) using a mixed design and t-tests were used to analyze differences between groups and changes across time. For the ANOVA, whenever an interaction was detected, separate ANOVAs for both group levels were conducted. Pearson correlation coefficient (r) was used to analyze correlations among theta, learning and the number of new neurons in the hippocampus. A regression analysis was applied in order to examine non-linear relationships among theta, learning and the number of BrdU positive cells in the hippocampus.

### Histology

Rats were sacrificed 21 days after the BrdU injection. This is a time point when many of the newly generated cells have already died, leaving those that were rescued by learning [23]. Rats were overdosed with an i.p. injection of sodium pentobarbital (Sleepaway, 26 mg/ml, Fort Dodge Animal Health, Fort Dodge, IA, USA) and intracardially perfused with 4% paraformaldehyde in 0.1 M phosphate buffer. Then, the brain was extracted, postfixed, and coronal sections (40 µm) cut through the entire DG of the left hemisphere using a vibratome. Every 12th unilateral section throughout the DG (6.3 to 1.8 mm posterior to bregma, see [24]) was collected and mounted on a slide. BrdU peroxidase staining was performed as described earlier (for protocol, see [7]). A cresyl violet counterstain was used. From the stained slides, estimates of total numbers of BrdU-labeled cells were obtained using a modified unbiased stereology protocol [6], [23], [25]. In essence, the number of BrdU-labeled cells in the granule cell layer and the hilus was counted and multiplied by 24 to obtain an estimate of the total number of BrdU-labeled cells in the hippocampi. Slides were coded and cell counting was conducted by an experimenter that was unaware of experimental conditions. Numerous studies from our group and others have verified that ∼80% of cells labeled with BrdU mature into neurons when assessed using neuronal markers doublecortin [23], [26], [27], NeuN [28]–[30] or TuJ1 [22], [29]–[31]. The right hemisphere was used to assess the location of the electrode tips. The tissue was sectioned (40 µm) and slices were mounted on slides and stained with cresyl violet. The location of the electrode tips was verified using light microscopy.

## Results

### Very-long delay eyeblink conditioning is easier to learn than trace eyeblink conditioning

**Figure 2:**
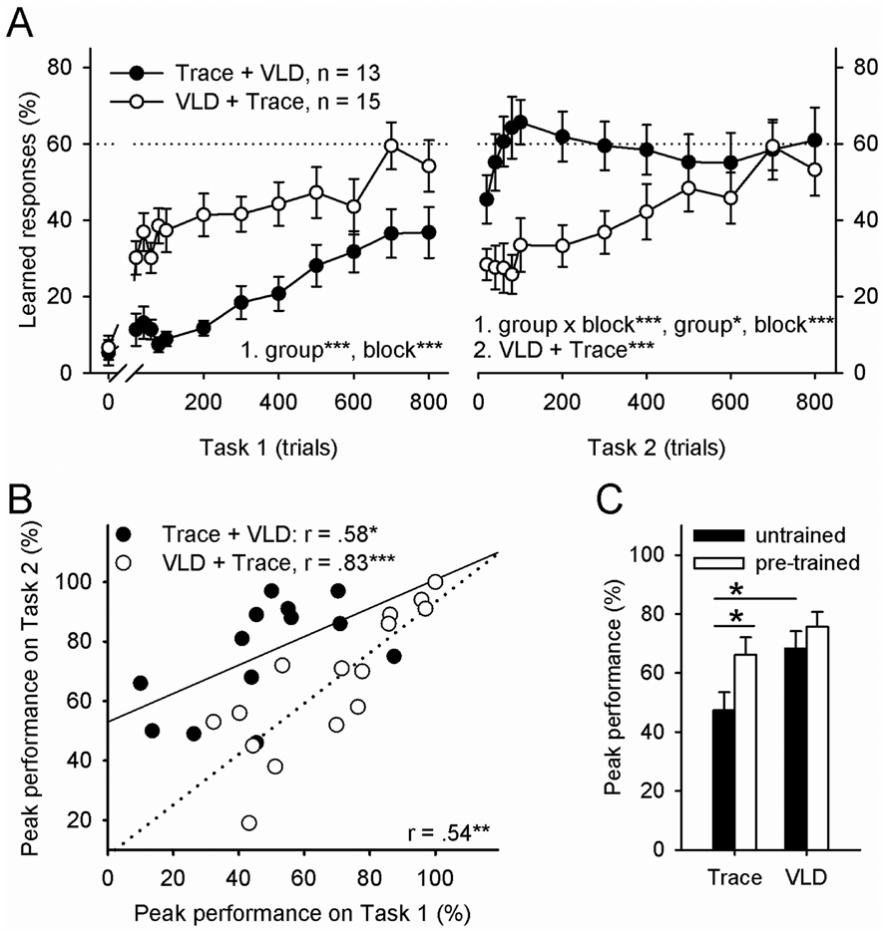
Learning was facilitated and predicted by previous learning of a similar task and very-long delay (VLD) conditioning was easier to learn than trace conditioning. In A learned responding is plotted in bins of 20 trials for the first 100 trials of each task and in bins of 100 trials from there on. The conditioned stimulus did not elicit eyeblinks before conditioning (Task 1 at 0). Learned responding increased during the initial training experience (Task 1), with elevated responding in animals trained with VLD conditioning compared to the group trained in trace conditioning. During training on the second task (Task 2), animals initially trained with the trace procedure rapidly acquired the VLD conditioned response whereas those initially trained with VLD required more trials to learn to time the conditioned response during the trace interval. Both groups learned to respond adaptively by the end of training (Task 2 at 800). B) Animals that learned well during the first task learned well during training on the second, related task. C) The highest percentage of learned responses attained during any given 100-trial block (Peak performance %) in the first phase of training (untrained) was significantly higher as a consequence of VLD than trace conditioning. When training was preceded by training on a similar task (pre-trained), the rats learned equally well both during VLD and trace conditioning. In A, statistically significant results of two-factor repeated measures ANOVAs are indicated (1.). If an interaction was detected, a separate ANOVA for each level was conducted (2.). Asterisks refer to statistical significance: * p<.05, ** p<.01, *** p<.001.

The behavioral results of the experiment are summarized in Figure 2. As illustrated in Figure 2A (Task 1 at 0), the CS did not elicit eyeblinks before training. During the first phase of training (see Fig. 2A Task 1), conditioned responding increased in response to both trace and very-long delay (VLD) conditioning (two-factor repeated measures mixed-design ANOVA, main effect of training block: F [11, 286] = 10.38, p<.001). However, conditioned responding was elevated in the group trained with VLD conditioning as compared to responding in the group trained with trace conditioning (main effect of group: F [1, 26] = 22.64, p<.001). Animals that emitted at least 60% learned responses during at least one 100-trial block were considered to have learned. Most (60%) of the rats trained with VLD task learned to emit an adaptive conditioned response, i.e. a blink that occurred within a 250 ms interval prior to the unconditioned stimulus. In contrast, only 23% of those trained with the standard trace conditioning task learned to emit the CR within a 250-ms period preceding the onset of the US. On average, by the end of training on task 1, rats trained with VLD emitted more learned responses than those trained with trace conditioning (Peak performance, i.e. highest percentage of CRs performed during any given 100-trial block, mean ± standard error of mean: 68±6 vs. 47±6), t (26) = 2.47, p = .02 (see Fig. 2C).

### Learning is facilitated and predicted by previous training on a similar task

In the second phase of training (see Fig. 2A Task 2), rats previously trained on one task were trained on the other task. An interaction of group and training block was evident (two-factor repeated measures mixed-design ANOVA, F [11, 286] = 4.22, p<.001). Also, significant main effects of group (F [1, 286] = 7.34, p = .012) and block (F [11, 286] = 4.22, p<.001) were present. Animals previously trained on trace conditioning readily learned to emit CRs during VLD conditioning. They expressed a rapid increase in the number of CRs during the first 100 trials, and their performance then plateau at about 60% CRs (one factor repeated measures ANOVA: F [11, 132] = 1.66, ns.). In contrast, animals that were initially trained with the VLD task had more difficulty learning the new task. Nonetheless, these animals did learn to emit CRs during the 250 interval before the US (one factor repeated measures ANOVA, main effect of block: F [11, 154] = 6.64, p<.001). Overall, they expressed robust learned responding by the end of the training experience.

Eighteen out of 28 rats (64% compared to 39% in the first task) learned the second task after having been trained on the first task. Thus, as expected, performance was facilitated after training on a similar task. Out of the 13 rats that were first trained with trace conditioning, 77% reached the learning criterion during training with VLD. In comparison, half (53%) the rats that were trained first with VLD learned the trace CR. During training on the second task, learning outcome no longer differed according to training protocol: Rats now trained on trace conditioning showed up to 66% (±6) CRs and those trained on VLD 76% (±5) CRs. Most importantly, the percentage of responses acquired during trace conditioning was elevated if the rats had been trained previously on VLD, at least as compared to rats that were not trained beforehand (66±6 vs. 47±6), t (26) = 2.18, p = .038 (see Fig. 2C). There was no equivalent difference for VLD conditioning (76±5 vs. 68±6) indicating that this task was learned equally well whether it was presented as a novel task (untrained) or as a task similar to previous experiences (pre-trained). Overall, the better an individual animal learned one task, the better it performed during training on the second task, r = .54, p = .003, n = 28 (see Fig. 2B). This was evident whether trace conditioning preceded VLD conditioning (r = .58, p = .039, n = 13) or vice versa (r = .83, p<.001, n = 15).

### Spontaneous hippocampal Type 2 theta activity predicts learning a novel task

**Figure 3:**
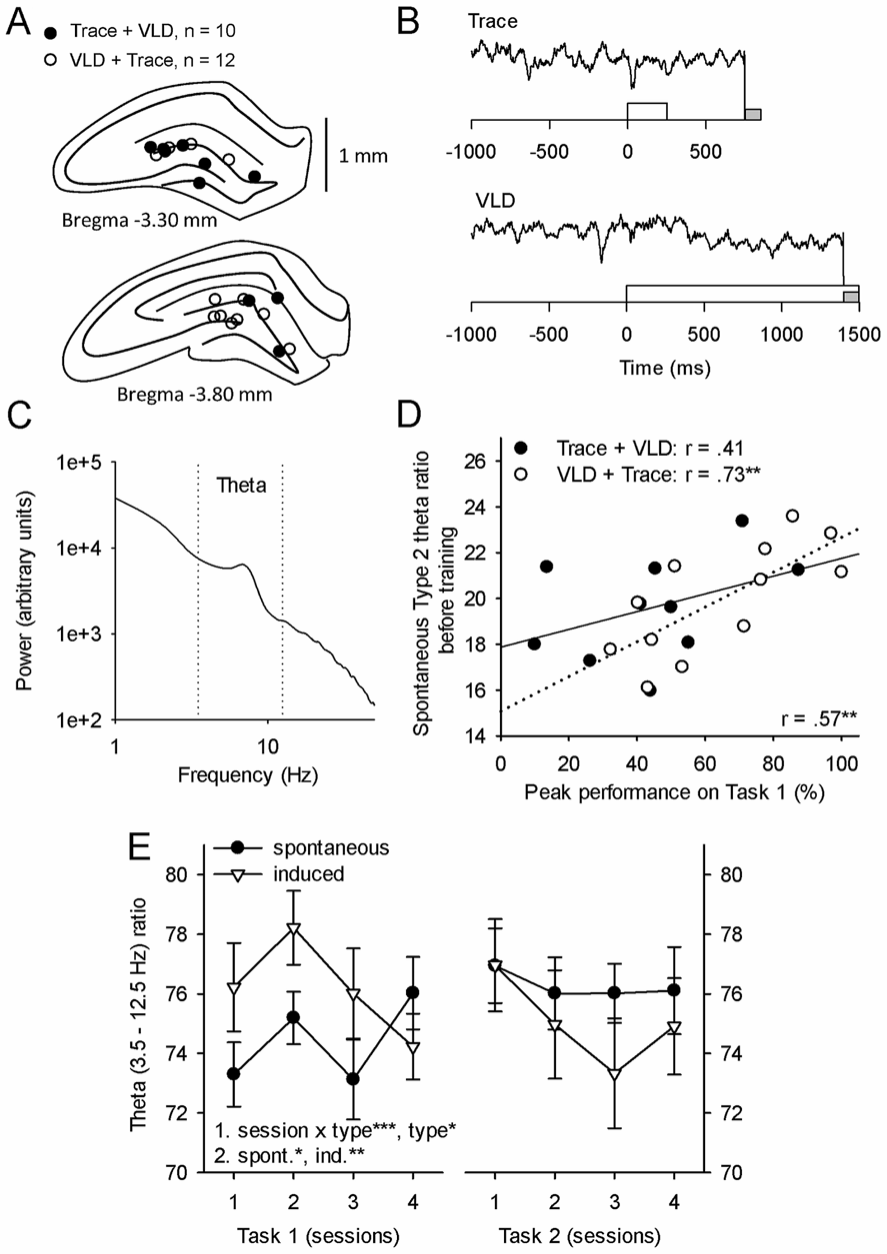
The relative amplitude of hippocampal theta activity predicted learning the first task and increased in response to the conditioned stimulus early in learning the first task. A) Twenty-two animals had correctly placed electrodes in the dentate gyrus. One electrode per animal was selected for analysis based on the location of the electrode tip. B) Representative examples of single-trial local-field potential (LFP) recordings from the dentate gyrus during the presentation of the conditioning stimuli. The white bar indicates the white-noise conditioned stimulus and the grey bar indicates the stimulation of the eyelid used as an unconditioned stimulus. C) Fast Fourier transform of spontaneous hippocampal LFPs illustrates a peak in power at the theta band (3–12 Hz). D) The relative power of spontaneous hippocampal Type 2 theta activity recorded prior to any training predicted learning the first task. E) The presentation of the conditioned stimulus induced a response within the theta-band early in training on the first task. In E, statistically significant results of two-factor repeated measures ANOVAs are indicated (1.). If an interaction was detected, a separate ANOVA for each level was conducted (2.). Asterisks refer to statistical significance: * p<.05, ** p<.01, *** p<.001.

Twenty-two rats had correctly placed electrodes in the dentate gyrus (Fig. 3A) and were used for the analyses regarding theta activity. Data from one electrode per animal was selected for analysis based on the location of the electrode tip. Representative examples of hippocampal recordings are presented in Figure 3B, and an average of the results of a FFT run on spontaneous hippocampal LFPs in Figure 3C. Type 2 theta ratio before training predicted performance during training on the first task (r = .57, p = .006, n = 22, see Fig. 3D). The correlation was only significant in those that were first trained with VLD. (r = .73, p = .007, n = 12). The correlation was of the same polarity but not significant in those that were initially trained with trace conditioning (r = .41, ns., n = 10). To conclude, rats that had high hippocampal Type 2 theta ratios prior to training learned the first task better.

In contrast to Type 2 theta, Type 1 theta did not correlate with performance on the first task (r = −.26, ns., n = 22). There was also no correlation between either type of theta and speed of learning during the first task: Trials to 10^th^ CR×Type 1, r = −.08, ns., n = 22; Trials to 10^th^ CR×Type 2, r = .07, ns., n = 22; Trials to criterion×Type 1, r = .13, ns., n = 8; Trials to criterion×Type 2, r = .21, ns., n = 8. In addition, no correlation was found between either type of theta recorded immediately before training on the second task and how well an animal learned that task (Type 1: r = −.11, ns., n = 22; Type 2: r = −.17, ns., n = 22). Also, no correlation was found between either type of theta recorded before training on the second task and how fast an animal learned that task: Trials to 10^th^ CR×Type 1, r = −.09, ns., n = 22; Trials to 10^th^ CR×Type 2, r = −.01, ns., n = 22; Trials to criterion×Type 1, r = .24, ns., n = 13; Trials to criterion×Type 2, r = −.04, ns., n = 13.

### Theta activity increases during training on a novel but not a similar task

Spontaneous and training-induced theta ratios were derived from the LFP data recorded immediately before and during each trial using FFT and are depicted across sessions in Figure 3E. Because of the nature of the data (stimulus-induced responses), the theta ratio was calculated for the whole band (3.5–12.5 Hz/1.5–12.5 Hz) and not separately for Type 1 and Type 2 theta bands (see Materials and Methods). The CS elicited an increase in the relative power of theta activity. This increase was only evident early during training on the firsts task (two-factor repeated measures ANOVA: interaction of recording type [spontaneous vs. induced] and session: F [3, 63] = 8.89, p<.001; main effect of recording type: F [1, 21] = 4.44, p = .047). Both spontaneous (one-factor repeated measures ANOVA, main effect of session: F [3, 63] = 3.74, p = .015) as well as CS-induced theta ratios (one-factor repeated measures ANOVA, main effect of session: F [3, 63] = 4.42, p = .007) revealed changes across training on the first task. For spontaneous theta, a cubic trend was significant (F [1, 21] = 10.58, p = .004) indicating an initial increase followed by a decrease and then another increase again across training on the first task. For the CS-induced theta a quadratic trend was significant (F [1, 21] = 6.23, p = .021) indicating an initial increase early in training and then a steady decrease towards the end of training on the first task. During training on the second task no significant changes across sessions or differences between ongoing and induced theta were present (main effect of session: F [3, 63] = 1.82, ns.; main effect of recording type: F [1, 21] = 0.95, ns.; interaction: F [3, 63] = 0.63, ns.).

### Learning a similar task still increases the survival of adult-born cells in the hippocampus

**Figure 4:**
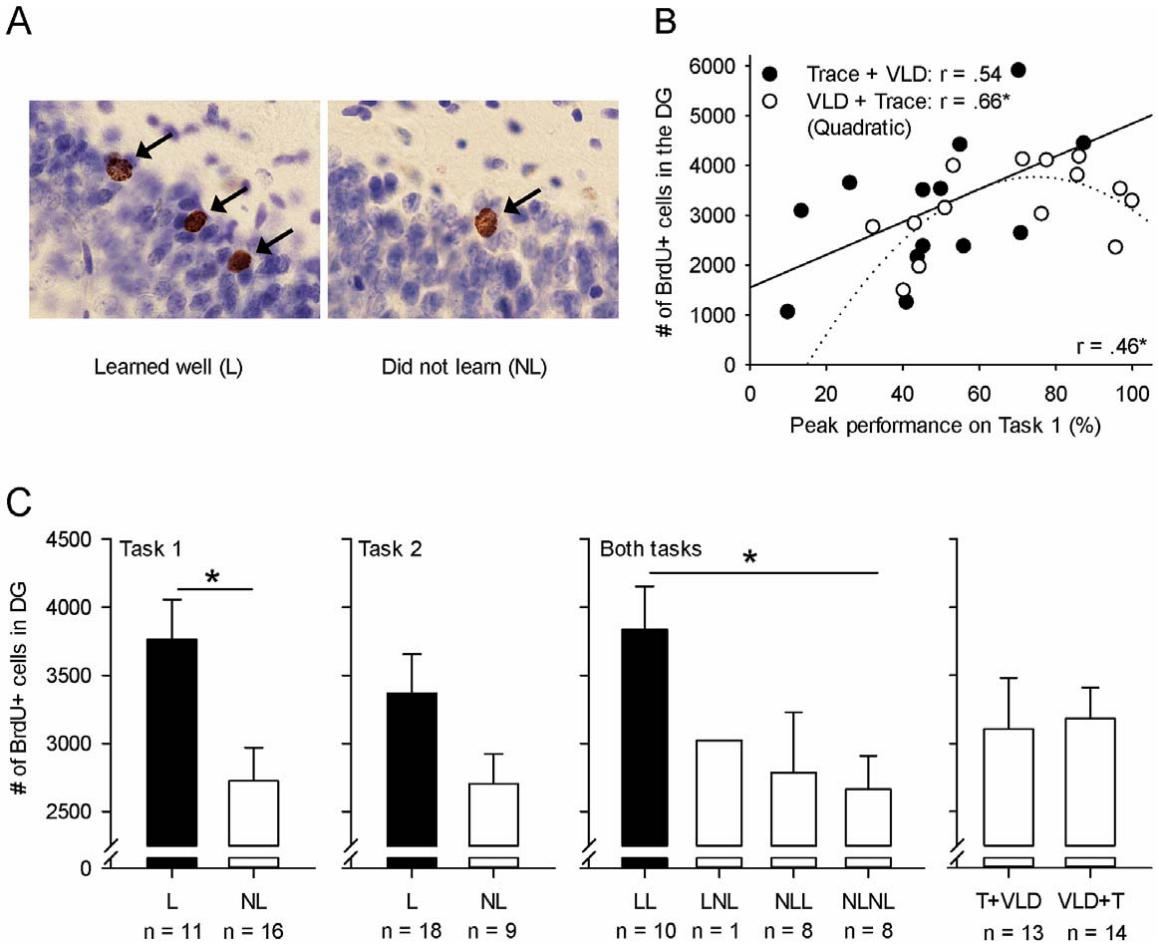
Learning predicted the number of BrdU positive cells that would survive in the dentate gyrus (DG) after all training. A) Representative examples of BrdU-positive cells from two animals: One that learned and one that did not learn. B) The better a rat learned the first task, the better it learned the second task and the more new cells resided in the dentate gyrus after all training had ceased. C) After training, animals were categorized into those that learned (L) and those that did not learn (NL). Those that learned either the first or both tasks well retained more new cells than those that did not learn or did not learn well. There was no difference in the number of BrdU positive cells present in the DG based on the order of the two tasks (Trace+VLD vs. VLD+Trace). Asterisks refer to statistical significance: * p<.05, ** p<.01, *** p<.001.

One rat was excluded from the data due to the complete absence of BrdU staining. Representative photomicrographs of cells in the granule cell layer of the DG labeled with BrdU are presented in Figure 4A. On average, 3147 (±210) BrdU positive cells were present in the DG 21 days after the BrdU injection. Recall that the injection was given after training on the first task had ceased, one week before training on the second task was started (see Fig. 1A). Overall, the better a rat learned the first task, the more immature neurons survived in response to training on the second task (r = .46, p = .016, n = 27) (see Fig. 4B). Note that these new cells were not yet generated when the animals were trained on the first task. For animals that were trained with VLD followed by trace, the relationship between performance and the number of surviving new cells was quadratic (regression analysis: r = .66, F [2, 21] = 4.31, p = .042), suggesting an inverse U-shape. Thus, it would indicate that more BrdU positive cells were present in animals that tended to learn well but did not master the timing of the response during training on the first task. In those that were first trained with trace conditioning, the correlation between learning the first task and the number of new cells was linear (r = .54, ns., n = 13) but not statistically significant. There was no difference in the number of BrdU positive cells based on the order of the tasks, t (25) = .18, ns. (Fig. 4C). Also, there was no observable relationship between the number of new cells present in the DG after training and how fast a rat learned either task (Trials to 10^th^ CR×cell count: Task 1: r = −.16, ns., n = 27 and Task 2: r = .05, ns., n = 27; Trials to criterion×cell count: Task 1: r = .38, ns., n = 11 and Task 2: r = .02, ns., n = 18) or how well a rat learned the second task (r = .38, ns., n = 27). Nor was there a correlation between either type of theta recorded prior to training on the second task and the number of BrdU positive cells in the dentate gyrus (Type 1: r = .29, ns., n = 22; Type 2: r = −.41, ns., n = 22).

To assess the relative effects of learning on the number of BrdU positive cells, we divided animals into those that learned (L) or did not learn (NL) either task. Subjects that learned the first task possessed significantly more adult-born cells in the DG after the experiment compared to rats that did not learn the first task, t (25) = 2.70, p = .012 (see Fig. 4C Learned: 3762±295 cells vs. Did not learn: 2723±246 cells). Subjects that learned the second task well also possessed more adult-born cells in the DG after the experiment compared to rats that did not learn. However, this effect was not statistically significant, t (25) = 1.53, ns. (see Fig. 4C Learned: 3369±286 cells vs. Did not learn: 2702±220 cells). Overall, animals that learned both tasks well (LL) retained more adult-born cells in the DG compared to rats that did not learn either task (NLNL), t (16) = 2.82, p = .012 (see Fig. 4C Learned: 3836±315 cells vs. Did not learn: 2662±245 cells).

## Discussion

Learning increases the survival of adult-born cells in the hippocampus that are approximately one week of age at the time of training (for review see [32]). How the new cells are rescued from death is not known and neither is their function once they have been incorporated into the existing circuitry of the hippocampus. Here we hypothesized that hippocampal theta activity (3–12 Hz) is necessary to rescue new cells from death in response to learning. We further speculated that new neurons that have been rescued from death by learning are used to learn related tasks in the future, and therefore learning a related task would not rescue more cells and would not induce theta activity. Congruent with the idea, we hypothesized that there is some upper limit on the number of new neurons that can be retained in response to repeated learning opportunities. To test these hypotheses, rats were trained on two hippocampus-dependent classical conditioning tasks that differed in temporal organization (stimulus duration and overlap) successively while recording hippocampal local-field potentials. Cells that were generated after training on the first task were labeled with BrdU. One week later, at a time when learning a novel task rescues new neurons from death [7], animals were trained on the second task. The number of cells that survived was assessed at a time when most of the new cells would have died in the absence of learning. The results of our experiment did not fully support our hypotheses: First, cells that were generated after learning one associative task were still more likely to survive after training on another, similar task, provided that learning occurred. Second, the increase in cell survival was unaccompanied by an increase in stimulus-induced theta activity. These results dissociate theta from the increased cell survival in response to learning. The most important observation is that new cells in the hippocampus were rescued from death in response to a relatively minor change in the task. Thus, there does not appear to be an upper limit on the number of cells that can be rescued from death – as long as animals learn and then learn again based on previous experiences across their lifetime.

As expected [33], [34], animals that learned the first task tended to learn better during training on the second, related task, and those that did not learn during the initial training experience did not perform well during training on the second task. Although the two training tasks manipulate the same conditioning stimuli, they nonetheless differ in a number of important ways. During trace conditioning, the animal learns to associate two stimuli separated by a temporal gap and learns to emit an eyeblink response during the gap, immediately prior to the US. During very-long delay conditioning, the animal is presented with a long-duration CS which overlaps and terminates with the US. Therefore, the two stimuli are contiguous in time and the animal must learn to withhold its response during most of the CS until just prior to the US. Both tasks are dependent on the hippocampus, although trace eyeblink conditioning is generally more difficult to learn than VLD eyeblink conditioning [21]. The difficulty in trace conditioning is presumably due to the fact that the animal must maintain a memory trace of the CS throughout the duration of a stimulus-free trace period to emit the response at the appropriate time. Regardless, the present data indicate that animals require more training trials to learn the trace response than to emit a similar number of CRs during VLD conditioning but both groups reach similar degrees of performance by the end of training. Note that the relatively low number of CRs presented here are also indicative of the criterion used to detect them: Conditioned responses are often defined as responses occurring during the entire duration of the trace period (500 ms) or an equivalent period preceding US onset (delay conditioning) whereas here we analyzed eyeblink responses that occurred within a 250-ms time period immediately preceding US onset. We used this more stringent criterion to detect individual differences in conditioned response acquisition and to correlate that with theta activity and the number of surviving cells in the dentate gyrus (see Materials and Methods).

The relative power of Type 2 theta activity recorded prior to any training correlated with individual differences in learning. This result is consistent with studies in rabbits. That is, animals with a high proportion of Type 2 theta activity prior to any training tend to learn faster [14] and better [15]. Similar correlations between baseline levels of theta activity and subsequent performance on learning tasks have also been found in humans [35], [36]. Based on these results, it can be proposed that prominent hippocampal Type 2 theta activity reflects an endogenous neural state which allows an animal to form associations between events that occur close in time. In contrast to previous studies, we did not observe a correlation between speed of learning and endogenous theta. However, this may simply reflect differences in species. Most studies of this sort were conducted in rabbits, which tend to express a slow consistent rate of acquisition, whereas this study used rats which can emit abrupt changes in learned responding across trials. Also, training was massed into 4 daily sessions of 200 trials each whereas studies with rabbits tend to present fewer trials per session spaced out over more days.

In addition to the correlation between spontaneous theta activity and subsequent learning, we also observed that theta activity increased in response to the CS during the learning process. The responses to the CS were within the Type 1 frequency-band but unaccompanied by motor activity and hence are referred to here as theta-band responses. Specifically, the CS induced an increase in the relative power of theta activity early in training on the first task. This is consistent with studies in rabbits, which report a strong correlation between theta-band oscillatory responses and learning early in training [14], [20]. Interestingly, the CS-induced increase in theta activity did not extend into training on the second task, even though animals still expressed considerable learning in terms of adjusting the timing of their response to match the temporal parameters of the new task (shaping the learned response, see [37]). Hence, it would appear that theta activity is preferentially induced when animals learn a new association between events but not when learning new configurations of those same (or similar) events. More generally, the changes in CS-induced theta-band activity during the successive training experiences reported here could reflect the degree to which the hippocampus is communicating with other brain regions during different phases of the learning process. In previous studies, it has been shown that, in response to the conditioning stimuli, hippocampal theta activity synchronizes with theta activity in the cerebellum [16], [17]. In addition, animals that learn fast exhibit stronger phase-locking of hippocampal theta activity in response to the CS early in training [20]. These findings together suggest that hippocampo-cerebellar communication at the theta frequency is strongest early in training when the association between the conditioning stimuli is first acquired. Later modifications of learned behavior in terms of timing might rely more on, for example, processing within the hippocampus [38], [39] and/or the cerebellar cortex [40]. On a behavioral level, it has been shown that pretraining on a delay conditioning task renders trace conditioning independent of the hippocampus [21]. Thus, it may be that the “pretraining” with the one task rendered the hippocampus (and theta oscillations within it) superfluous when it came to learning the second task.

Our hypothesis had been that learning during one associative memory task would enhance the survival of one cohort of new neurons and those new neurons would then mature and be used to learn a second, similar task. As a consequence, we anticipated that the cells would not be affected by the second learning experience, i.e. that there would be no difference in cell counts between animals that learned and did not learn. This hypothesis was further fueled by previous studies [7] indicating simply expressing or reinstating a memory is not sufficient to rescue new cells. Indeed, we further anticipated that if any, it would be the animals that did *not* learn the first task but *did* learn the second task that would show higher numbers of BrdU positive cells in the hippocampus after the experiment. However, contrary to our hypothesis, animals that acquired a robust learned response during training on the first (and the second) task retained more new cells than animals that did not learn. Thus, it seems that learning one task will enhance learning a related task in the future and this process will in turn increase the number of surviving cells. That said, it is noted that many types of learning processes, such as maze training with a visible cue [6], short delay eyeblink conditioning [6], [23], short-trace eyeblink conditioning [41] or trace conditioning if the animals have already learned the association during short-delay conditioning [22], do not increase cell survival. Therefore, the cells are not “indiscriminate” in their response to learning. Rather, they are especially responsive to a select group of learning processes, including the classical conditioning procedures of trace and very long delay conditioning. As demonstrated here, new cells are responsive to these processes, even if born into a hippocampus with a history of very similar learning experiences.

It is important to emphasize that learning increases the *survival* of cells that are already present and about one week of age at the time of training, and does not increase the number of cells that are generated at the time or right after training [7]. Also, the number of cells that are being generated during the training period does not correlate with the percentage of learned responses emitted during that training period ([7], Figure S1). Therefore, unlike endogenous theta activity, which predicts performance before any training occurs, the number of new cells present before training does not correlate with performance once it occurs [7], [9]. Of course, there are many manipulations that can cause drastic changes in cell proliferation, which then can relate to learning, but this is another issue. That said, it is likely that “smart” animals will have retained more new neurons as a consequence of learning well throughout their lives and as a consequence should have more mature neurons in their hippocampus. The issue is similar to that reported for London taxi drivers: Experienced taxi drivers have an enlarged dorsal hippocampus [42], presumably because of their vast experience with spatial navigation, rather than some innate ability [43]. To conclude, as far as we can extrapolate, the connection between learning and the number of immature neurons surviving in the hippocampus reported here is not attributable to environmental or innate processes (such as the degree of cell proliferation) that occurred prior to the training experience. If they were, then our previous manipulations with short-delay [6], [23], short trace [41], visible platform maze training [6], unpaired training [8], etc. would have elicited the same response as that seen in animals that were trained with trace or very long delay conditioning (i.e. an increase in cell survival). Rather, these results indicate that the act of learning rescues new neurons from death and new cells continue to be rescued with each new learning experience, even if the learning is based on previously-acquired information.

As discussed, we had hypothesized that increased electrophysiological oscillatory activity within the theta band could contribute to recruiting new cells into functional networks within the hippocampus during learning. However, as discussed above, hippocampal theta activity only increased during training on the first task and not during training on the second task, despite clear evidence of learning and enhanced cell survival. These results dissociate induced theta activity from the increase in cell survival in response to learning. Moreover, these results indicate that theta activity is engaged only early in training, when learning a novel association whereas new neurons remain responsive to learning tasks similar to previous experience. It is important to note that these results do not indicate (or test for that matter) whether new hippocampal cells are necessary for learning temporal relationships and/or differences in stimulus contiguity but rather indicate that they respond to learning about changes in the temporal organization of external events and survive as a result.

In conclusion, the present data indicate that hippocampal theta activity predicts and may participate in forming associations between novel, related events in the environment. However, once the association is formed, theta activity is not increased during training, even as new temporal relationships are learned. In contrast, each new experience can apparently rescue a new cohort of cells from death, provided that learning occurs. Therefore, theta is predictive of performance during a new learning experience whereas the new neurons survive as a consequence of that learning and future perturbations of that learning. These results place theta activity and newly generated neurons in a context of continuous learning, one that remains responsive to an ever-changing environment and builds on past experience for survival.

## Supporting Information

Figure S1 **Cell proliferation in the dentate gyrus does not correlate with learning.** To assess whether cell proliferation correlates with learning, a subset of data previously published by Anderson et al. [7] was further analyzed. There was no correlation between how many new cells were generated in the hippocampus immediately prior to and during training and how well an animal learned trace eyeblink conditioning (r = .05, ns., n = 14).
(TIF)
