# Supplementary figures and images for: Learning to Learn: Theta Oscillations Predict New Learning, which Enhances Related Learning and Neurogenesis
Source: PLoS One. 2012 Feb 10;7(2):e31375. doi: 10.1371/journal.pone.0031375 (PMC3277498; doi:10.1371/journal.pone.0031375)

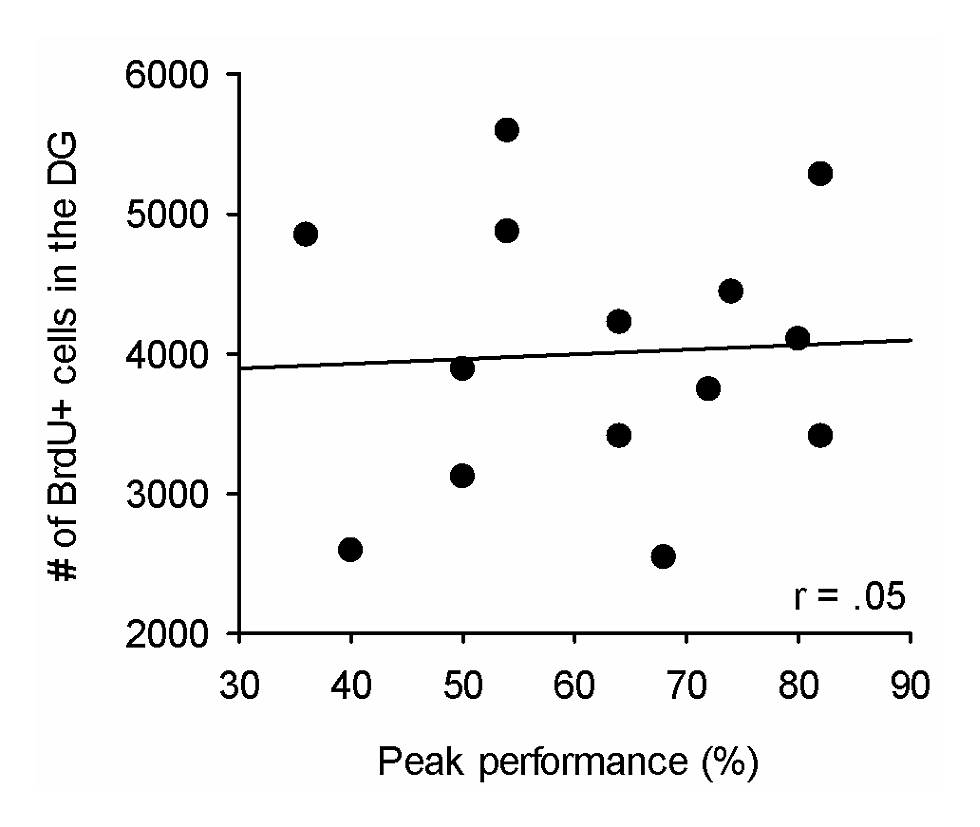

Supplement: Figure S1 — Cell proliferation in the dentate gyrus does not correlate with learning. To assess whether cell proliferation correlates with learning, a subset of data previously published by Anderson et al. [7] was further analyzed. There was no correlation between how many new cells were generated in the hippocampus immediately prior to and during training and how well an animal learned trace eyeblink conditioning (r = .05, ns., n = 14). (TIF) [file pone.0031375.s001.tif]
